# A distinct case of an 8-year-old female with cyclic neutropenia presenting with *C. septicum* abdominal sepsis and myonecrosis requiring a bowel resection and leg fasciotomy

**DOI:** 10.1093/jscr/rjad512

**Published:** 2023-09-18

**Authors:** Harrison Anzinger, Lina Cadili, Amanda Li, Amanda Barclay, Allen H Hayashi

**Affiliations:** Department of Pediatrics, Faculty of Medicine, University of British Columbia, Vancouver, British Columbia V6H OB3, Canada; Division of General Surgery, Department of Surgery, Faculty of Medicine, University of British Columbia, Vancouver, British Columbia V5Z 1M9, Canada; Department of Pediatrics, Faculty of Medicine, University of British Columbia, Vancouver, British Columbia V6H OB3, Canada; Department of Pediatrics, Faculty of Medicine, University of British Columbia, Victoria, British Columbia V8Z 6R5, Canada; Division of General Surgery, Department of Surgery, Faculty of Medicine, University of British Columbia, Vancouver, British Columbia V5Z 1M9, Canada; Division of Pediatric Surgery Island Medical Program, Vancouver Island Health Authority, Victoria, British Columbia V8Z 6R5, Canada

**Keywords:** pediatric surgery, pediatrics, cyclic neutropenia, Clostridium septicum, myonecrosis, sepsis

## Abstract

*Clostridium septicum* is a very rare cause of severe spontaneous pediatric enterocolitis and is often associated with underlying malignancy or immunocompromise. Likewise, cyclic neutropenia is a rare congenital immunodeficiency that is characterized by cyclical periods of neutropenia, often with more severe symptoms in the pediatric population. Here, we present a unique case of spontaneous *C. septicum* enterocolitis, sepsis, and myonecrosis in a child with undiagnosed cyclic neutropenia. Early recognition of pediatric sepsis, frequent reevaluation and identification of rapidly progressive infection, and early surgical intervention are critical for the effective management of a rare and severe infection.

## Introduction

*Clostridium septicum* is a gram-positive, anaerobic bacillus which rarely causes pediatric enterocolitis and myonecrosis, which can rapidly progress and lead to serious morbidity and mortality [1]. While commonly associated with trauma, *C. septicum* infections can occur spontaneously and are often associated with an underlying malignancy or immunodeficiencies [1–3]. While spontaneous cases often start in the abdomen, dissemination across the body is common, often involving extensive myonecrosis at distal sites. Mortality rates in pediatric cases are estimated at 57% [1]. Here, we present a rare case of *C. septicum* infection in an 8-year-old female with undiagnosed primary immunodeficiency.

## Case report

**Figure 1:**
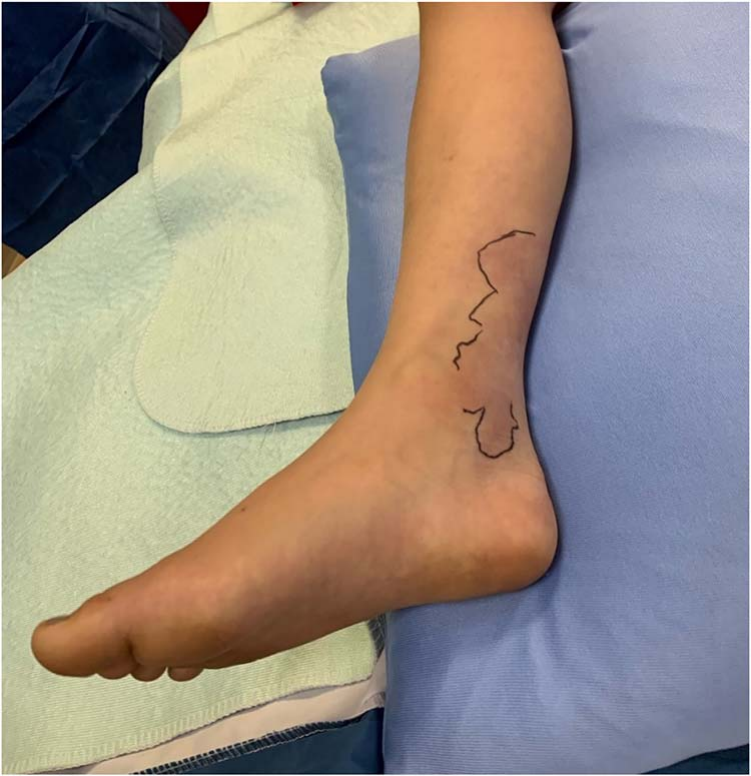
The patient’s right leg upon presentation to the tertiary care hospital, with the area of erythema and edema outlined with a skin marker.

**Figure 2:**
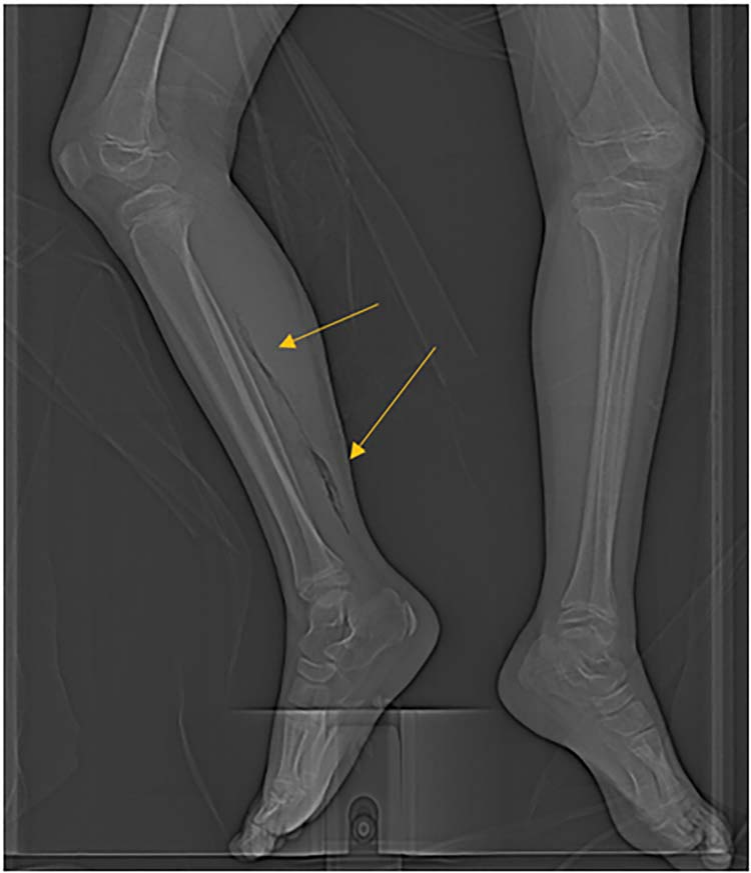
CT scan demonstrating extensive air in the soft tissue of the right leg.

**Figure 3:**
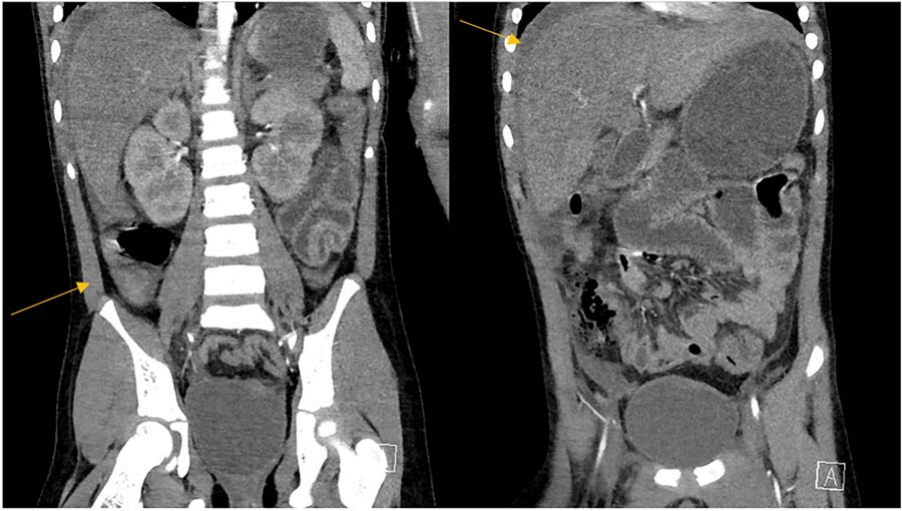
Abdominal CT scan demonstrating intrabdominal free fluid and a collection in the right lower quadrant.

The patient was an 8-year-old female who presented to hospital with a 3-day history of fever, abdominal pain, and coffee ground emesis. On presentation, she was afebrile, tachycardic, and hypotensive. On examination, she had an acute abdomen and septic shock. Initial laboratory investigations revealed a venous pH of 7.08, CO_2_ of 47 mmHg, bicarbonate of 14 mmol/L, lactate of 3.9 mmol/L, white blood cell count of 3.7 × 10^9^/L with an absolute neutrophil count of 0.96 × 10^9^/L, C-reactive protein of 389 g/L, INR of 1.7, PTT 36 s, fibrinogen of 5 g/L, and an acute kidney injury with a creatinine of 100 micromoles/L and urea of 10 mmol/L. An abdominal ultrasound revealed a starry sky appearance of the liver with moderate free fluid in the right upper quadrant. She was not fluid-responsive and thus was treated with an epinephrine infusion and empiric antimicrobial therapy with ceftriaxone and vancomycin. Transportation was arranged to a tertiary care center with access to pediatric intensive care and surgical services. In transport she developed mild erythema to the right calf with severe pain to palpation and dorsiflexion out of proportion of clinical findings (Fig. 1). Her creatine kinase level was 10 000 U/l and a computerized tomography (CT) scan of the right leg demonstrated extensive soft tissue gas and myonecrosis (Fig. 2). An abdominal CT scan demonstrated evidence of bowel perforation in the right lower quadrant with associated loculation, intra-abdominal free air and fluid, and proximal bowel dilation (Fig. 3). Considering these findings, antibiotics were broadened to piperacillin-tazobactam, vancomycin, and clindamycin, and she was urgently taken to the operating room.

**Figure 4:**
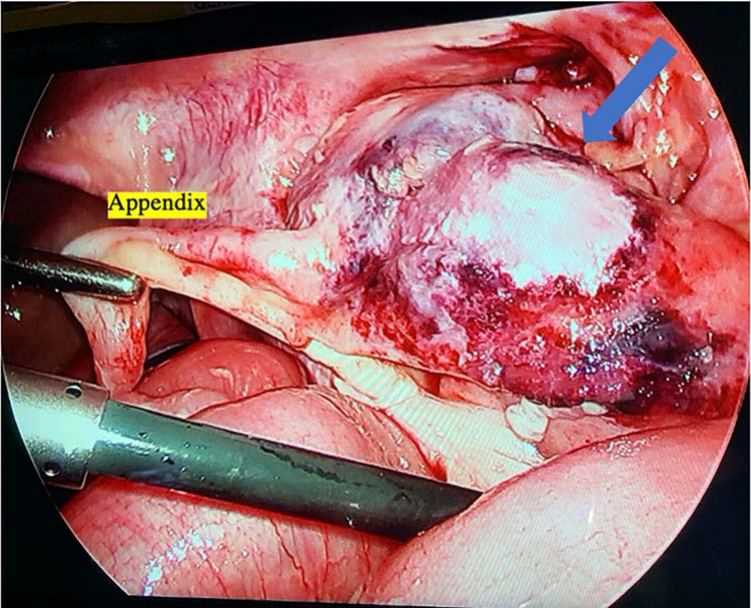
Intraoperative photograph of the ileocecal junction with extensive inflammation and necrosis.

Right leg fasciotomy and debridement were performed by an orthopedic surgeon; there was evidence of ischemia to the distal gastrocnemius. A diagnostic laparoscopy was then performed, which revealed a normal liver and extensive adhesions. She was found to have free fluid and an ischemic terminal ileum, which extended to the cecum and ascending colon (Fig. 4). Given these findings and her clinical status, an ileocolic resection with an end ileostomy was performed. Postoperatively, she returned to the Pediatric Intensive Care Unit. Her intra-abdominal fluid and deep leg swab cultures were positive for *C. septicum*, and her antibiotics were switched to ceftriaxone and metronidazole. She improved and was gradually weaned off ventilatory and cardiovascular supports. She returned to the operating room for closure of her fasciotomies 3 days following her initial surgery. She required three more surgical debridements of her right leg over the course of the next 2 months of her admission.

During her admission following her acute illness, she demonstrated repeat cycles of neutropenia every 3–4 weeks with ANC nadir of 0 × 10^9^/L. Further review of her medical history was notable for two previous admissions to hospital for IV antibiotics, previous ANC of 0.32 × 10^9^/L and 0.57 × 10^9^/L during separate febrile illnesses, extensive dental carries, and frequent oral sores occurring every 2–3 months lasting 1–2 weeks. Considering this history of neutropenia, and symptoms consistent with previous recurrent neutropenia, concern was raised for cyclic neutropenia as her underlying diagnosis. Genetic testing confirmed this diagnosis. She remains admitted and continues to improve with rehabilitation.

## Discussion

Cyclic neutropenia has a documented association with *C. septicum* infections and is characterized by recurrent neutropenia typically in 3-week intervals [1, 2, 4]. Children affected by this condition tend to have more severe symptoms than adults, typically characterized by recurrent episodes of oral ulcerations, gingivitis, lymphadenopathy, fever, tonsilitis, fatigue, or skin infections five or more times per year [5]. Cyclic neutropenia is caused by a mutation of the *ELANE* gene that encodes for neutrophil elastase, resulting in cytotoxic neutrophil esterase being packaged improperly, causing increased cell death [6]. Treatment typically involves granulocyte colony stimulating factor injections to lessen the degree of neutropenia in addition to close observation and early treatment of infection [6].

At presentation, our patient’s initial ultrasound results demonstrated a starry sky appearance of the liver. Starry sky, also known as periportal hyperechogenic, is a nonspecific finding of abdominal inflammation that is more prominent in pediatric patients [7, 8]. The differential is broad but includes a wide range of pro-inflammatory intra-abdominal processes, including but not limited to hepatitis, appendicitis, terminal ileitis, intussusception, gastroenteritis, mesenteric lymphadenitis, and inflammatory bowel disease.

This rare case demonstrates the importance of careful physical examination and frequent reassessment in the setting of a rapidly evolving infection. Recognition of pediatric sepsis and timely antibiotic administration in her rural community slowed the progression of her disease. Despite medical management, right leg myonecrosis developed rapidly over the course of a few hours during transport, and prompt recognition on arrival allowed timely diagnostic evaluation via CT scan and immediate surgical intervention for definitive source control. Failure to quickly recognize the severity and rapid progression of infection would have likely led to severe morbidity or death. Following stabilization, careful and detailed history allowed for recognition of symptom constellation that resulted in her cyclic neutropenia diagnosis.

## Data Availability

There is no data to provide for this case report. We cannot provide access to patient data for confidentiality reasons.
